# MicroRNA Expression Profiling Predicts Nodal Status and Disease Recurrence in Patients Treated with Curative Intent for Colorectal Cancer

**DOI:** 10.3390/cancers14092109

**Published:** 2022-04-23

**Authors:** Matthew G. Davey, Gerard Feeney, Heidi Annuk, Maxwell Paganga, Emma Holian, Aoife J. Lowery, Michael J. Kerin, Nicola Miller

**Affiliations:** 1 Department of Surgery, Lambe Institute for Translational Research, National University of Ireland, H91 YR71 Galway, Ireland; g.feeney3@nuigalway.ie (G.F.); heidi.annuk@nuigalway.ie (H.A.); aoife.lowery@nuigalway.ie (A.J.L.); michael.kerin@nuigalway.ie (M.J.K.); nicola.miller@nuigalway.ie (N.M.); 2 School of Mathematical and Statistical Sciences, National University of Ireland, H91 H3CY Galway, Ireland; m.paganga1@nuigalway.ie (M.P.); emma.holian@nuigalway.ie (E.H.)

**Keywords:** colorectal cancer, miRNA, genomics, cancer diagnostics, personalised medicine

## Abstract

**Simple Summary:**

Determining the degree of nodal involvement provides key prognostic information in several malignancies, including colorectal cancer (CRC). Furthermore, predicting long-term outcomes in such cancers often proves challenging to the multidisciplinary team. Therefore, the purpose of this translational research study was to evaluate the role of mi(cro)RNAs as biomarkers used to predict nodal status and recurrence in patients being treated for CRC. This analysis involved the quantification of miRNA targets in 74 patients with CRC using real-time reverse transcriptase polymerase chain reaction. Aberrant expression of miR-21 and miR-135b correlated with increased metastatic disease in local lymph nodes following resection. Interestingly, increased expression of miR-195 displayed strong capabilities of predicting the time to disease recurrence. These results add to the growing evidence illustrating the value of using miRNA expression profiling to inform patient outcomes in cancer. These findings may be further validated in further studies as we attempt to personalise the management paradigm for prospective patients diagnosed with CRC.

**Abstract:**

**Background:** Approximately one-third of colorectal cancer (CRC) patients will suffer recurrence. MiRNAs are small non-coding RNAs that play important roles in gene expression. We aimed to correlate miRNA expression with aggressive clinicopathological characteristics and survival outcomes in CRC. **Methods:** Tumour samples were extracted from 74 CRC patients. MiRNAs were quantified using real-time reverse transcriptase polymerase chain reaction. Descriptive statistics and Cox regression analyses were performed to correlate miRNA targets with clinicopathological and outcome data. **Results**: Aberrant miR-21 and miR-135b expression correlate with increased nodal stage (*p* = 0.039, *p* = 0.022). Using univariable Cox regression analyses, reduced miR-135b (β-coefficient −1.126, hazard ratio 0.324, standard error (SE) 0.4698, *p* = 0.017) and increased miR-195 (β-coefficient 1.442, hazard ratio 4.229, SE 0.446, *p* = 0.001) predicted time to disease recurrence. Survival regression trees analysis illustrated a relative cut-off of ≤0.488 for miR-195 and a relative cut-off of >−0.218 for miR-135b; both were associated with improved disease recurrence (*p* < 0.001, *p* = 0.015). Using multivariable analysis with all targets as predictors, miR-195 (β-coefficient 3.187, SE 1.419, *p* = 0.025) was the sole significant independent predictor of recurrence. **Conclusion**: MiR-195 has strong value in predicting time to recurrence in CRC patients. Additionally, miR-21 and miR-135b predict the degree nodal burden. Future studies may include these findings to personalize therapeutic and surgical decision making.

## 1. Introduction

Colorectal carcinoma (CRC) is the second highest cause of cancer-related mortality in the western world with over one million new diagnoses each year [1,2]. Traditionally, outcomes for those diagnosed with CRC were poor [3], with survival rates expected to be less than 50% at five years [4]. In recent years, we have observed an improvement in the anticipated oncological and survival outcomes for these patients [5], which coincides with a number of notable advances in the detection and management of the disease [6,7]. These advances include the increased availability and uptake in colonoscopy screening programmes, more refined surgical approaches to cancer resection, as well as the use of multimodal therapeutic strategies in a multidisciplinary approach to personalized care [8,9]. Despite these marked improvements in clinical outcomes for a significant proportion of patients, approximately 17–35% of those who are successfully treated with curative intent unfortunately will succumb to disease recurrence [10,11] and prognoses in the setting of CRC relapses are very poor [12,13]. Additionally, identifying those at an increased risk of relapse proves extremely challenging to the oncologist as disease recurrence is typically unpredictable. Therefore, the modern translational research paradigm has evolved, placing a large emphasis on biomarker discovery programmes aiming to identify novel biomarkers capable of rivalling current conventional biomarkers used to clinically predict relapse risk (i.e., extramural vascular invasion (EMVI), tumour budding, and carcinoembryonic antigen (CEA), etc.,) [14,15,16]. Despite rigorous experimentation, there have been only modest results supporting the utility of surrogate biomarkers capable of predicting disease recurrence in patients with locally advanced CRC [17]. 

Mi(cro)RNAs are small (19–25 nucleotides in length) endogenous non-coding ribonucleic acids (RNA) that are understood to play important regulatory roles in governing gene expression. These molecules achieve this through binding to 3′ or 5′ untranslated regions of target messenger RNA (mRNA) at a post-transcriptional level, which directly impacts gene expression through increased or inhibitory effects on mRNA profiles [18,19]. Aberrant miRNA expression profiles have been observed in a diversity of pathological processes, including cancer development, metastases, and disease recurrence [20], and there has been an increasing realisation that miRNA profiling may be useful in patient prognostication [18]. Furthermore, it is now well recognized that miRNAs remain stable in a large proportion of biological tissues (including tumour tissue, ‘normal’ epithelium, and circulation) and may be quantified relatively simply and inexpensively using real-time quantitative reverse transcriptase polymerase chain reaction (RT-qPCR) methodology [21,22,23].

In recent times, the efforts of oncological and translational research have focused on gauging response to current conventional therapies [24,25], providing patient-specific prognostication [18], and predicting survival in common malignancies, including CRC [19,21,24,25]. In CRC, miRNAs have been illustrated to have a dual role in oncogenesis, including cancer acceleration through oncomiR activation and tumour-suppressor miRNAs [26,27]. Therefore, discovering such clinically relevant biomarkers remains paramount in efforts to further personalise oncological treatment, particularly in the setting of ‘high-risk’ cases such as those identified to be at an increased risk of relapse by the CRC multi-disciplinary team. Accordingly, the primary aim of the current study was to correlate miRNA expression with aggressive clinicopathological tumour characteristics and to determine whether miRNA profiling is predictive of oncological and survival outcomes in a cohort of 74 patients diagnosed and treated with curative intent for locally advanced CRC disease. This involved the investigation of a panel of six miRNA expression targets in CRC tumour tissues from 74 patients using RT-qPCR. 

## 2. Materials and Methods

Following informed and written consent, CRC tumour tissue samples were obtained from 74 patients being treated with curative intent for CRC at Galway University Hospitals (GUH), which were biobanked at the Cancer Biobank at the Department of Surgery at National University of Ireland, Galway (NUIG). GUH is a large tertiary referral centre for cancer treatment serving the population of the west and north-west of Ireland. All patient demographic, clinicopathological, and survival data were obtained and updated within a prospectively maintained database.

### 2.1. Patient Workup and Colorectal Cancer Staging

All 74 included patients had previously presented to GUH for colorectal cancer in the west of Ireland for multidisciplinary management of their colorectal primary cancer. All patients had histopathological confirmation of CRC, which was confirmed at the local accredited histopathological laboratory. CRC was staged in accordance with tumour, nodes, and metastasis (TNM) staging system as outlined by the American Joint Committee on Cancer (AJCC) Version 8 [28]. Conventional immunophenotypical staining was performed using cytokeratin (CK) 20 (positivity indicating CRC) and CK7 (negativity indicating CRC) to discriminate adenocarcinoma from other histological colorectal subtypes [29]. Application of CDX2 staining was used to determine differentiation [30]. Tumour lymphatic invasion was evaluated using D2-40 staining and vascular invasion using CD34 (combined LVI) [31,32]. Tumour perineural invasion (PNI) was evaluated using S-100 staining [33] and extramural vascular invasion (EMVI) was assessed using elastin staining [34]. Radiological staging was performed using commuted tomography (CT) in all cases, with rectal cancers requiring additional pelvic magnetic resonance imaging (MRI). Radiological staging was performed using Siemens Somatom Definition AS 128 Slice CT scanners, while pelvic staging was evaluated in cases of rectal cancer using a short bore 1.5 T magnet (Magnetom Espree 1.5 T, Siemens Healthcare, Erlangen, Germany). Clinicopathological and survival data for the 74 included patients are outlined in Table 1.

### 2.2. MiRNA Targets 

Initial literature review identified a panel of 15 miRNA that were selected (miR-17, miR-20a, miR-21, miR-31, miR-132, miR-135b, miR-139-5p, miR-145, miR-148a, miR-150, miR-155, miR-195, miR-200c, miR-203, and miR-215) based on their previously reported relevance to CRC as well as other epithelial cancers. Then, based on the amplification profiles in one third of tissues (*n* = 27) as well as their previously reported relevance to CRC and other epithelial cancers in forming prognoses and outcome [21,25,35,36,37,38], we refined the panel to 6 miRNA (miR-21, miR-31, miR-135b, miR-150, miR-155, and miR-195) which were amplified in this study. The relevance of these miRNA to CRC disease from previous reports are outlined in Table 2. 

### 2.3. RNA Isolation and Storage

RNA was extracted using MagNA Pure Isolation (Roche) extraction process in accordance with manufacturers’ instructions. Total RNA was extracted from 1μL of homogenate CRC tumour tissue, respectively, with RNA concentrations and integrity determined using Denovix NanoDrop© spectrophotometry (Nanodrop ND-1000 Technologies inc., Wilmington, DE, USA) and an Agilent Bioanalyser (Agilent Technologies, Germany). RNA concentrations and associated 260/230 and 260/280 ratios were recorded (all within target range 2.0–2.2). Integrity was assessed using RNA 6000 Nano LabChip Series II Assays (for small RNA) on a 2100 Bioanalyzer (Agilent Technologies, Waldbronn, Germany). RNA was then transferred to storage tubes, labelled, and stored at −70 °C in the Cancer Biobank at the Department of Surgery at NUIG.

### 2.4. Analysis of miRNA Gene Expression

RNA samples underwent reverse transcription using primers specific to each specific miRNA target. RT-qPCR was carried out using TaqMan© assays (Applied Biosystems, Foster City, CA) in accordance with the manufacturer’s instructions. This involved 5 ng of tumour/Tan total RNA being reverse transcribed using the MultiScribe^™^ based High-Capacity cDNA Archive kit (Applied Biosystems), with reverse transcriptase controls included in reactions. Polymerase chain reactions (PCR) were conducted in final volumes of 10 μL using QuantStudio 7 Flex Fast Real-Time PCR System (Applied Biosystems). Briefly, reactions consisted of 1.0 μL cDNA, 5 μL TaqMan^®^ Universal PCR Fast Master Mix, 3.5 μL of nuclease-free water, and 0.5 μL TaqMan^®^ primer–probe mix (Applied Biosystems). Reactions were initiated with a 10-min incubation at 95 °C followed by 40 cycles at 95 °C for 15 s and 60 °C for 60 s. We utilised miR-26b as an inter-assay control derived from a breast cancer cell line (MDA-MB-468) that was included on each plate and all reactions were performed in triplicate. Based on previous work from our laboratory, miR-16 and miR-345 were selected as endogenous controls to standardize miRNA expression [39]. The threshold standard deviation (SD) for intra-assay and inter-assay replicates was 0.3. The percentage PCR amplification efficiencies (*E*) for each assay were calculated using the slope of the semi-log regression plot of cycle threshold vs. log input of cDNA (10-fold dilution series of five points) with the following equation and a threshold of 10% above or below 100% efficiency was applied: *E* = (10^−1/slope^ − 1) × 100. Thereafter, miRNA expression levels were calculated using QbasePlus software (Biogazelle, Gent, Belgium) using the geNorm method in order to ensure results were calibrated and normalised before being relatively quantified compared to the endogenous controls (miR-16 & miR-355) [39,40]. 

### 2.5. Statistical Analysis 

The Shapiro–Wilk test was used to determine distribution of the miRNA target expression values indicating non-normality. The Kruskal–Wallis test was applied to assess whether distributions were associated with aggressive clinicopathological parameters. Univariable and multivariable Cox regression analyses for time to recurrence were performed using both clinicopathological predictors and miRNA expression profiles in order to determine the added value of such miRNAs in predicting survival outcomes and recurrence. Cox regression results were expressed as β-Coefficient and exp(β-Coefficient), i.e., hazard ratio, with associated SE, 95% confidence intervals (CIs), and *p*-values. Regression trees were used to classify the patients by significantly relevant cut-offs for each miRNA. Receiver operating characteristic (ROC) curves were generated using binary logistic regression analysis with area under the curve (AUC), sensitivity, and specificity from non-crossover models expressing diagnostic test accuracy to inform survival and recurrence. All tests of significance were 2-tailed, with *p* < 0.050 indicating statistical significance. Statistical analysis was performed using the statistical package for social sciences (SPSS) Version 26.0 (Chicago, IL, USA) and R Version 3.2.3 (Boston, MA, USA).

### 2.6. Patient Follow-Up and Definitions

Patient follow-up was recorded through a prospectively maintained database; median lengths of follow-up were calculated using the reverse Kaplan–Meier method [41]. Data was obtained from a prospectively maintained institutional database. All data was cross-referenced with patient electronic and medical records. We defined recurrence as ‘suffering local or distant relapse of the invasive cancer following treatment with curative intent for CRC’. Disease-free survival (DFS) was defined as ‘freedom from local or distant relapse of the invasive cancer or death from any cause following treatment with curative intent for CRC’. Overall survival (OS) was defined as ‘freedom from mortality from any cause including and not limited to CRC’.

## 3. Results

### 3.1. Included Colorectal Cancer Patients

In total, 74 patients diagnosed with colorectal cancer who donated tumour tissue were included in this study. The mean age of the 74 included patients was 67.8 years (standard deviation ±12.5 years, range 38–90 years). Overall, 68.9% were male patients (51/74) and 70.3% were rectal carcinoma (52/74). The median follow-up was 85.6 months (± 9.5 months). The observed clinicopathological and survival statistics for these patients is included in Table 1. 

### 3.2. Associations between Clinicopathological Characteristics and miRNA Expression Profiles

**Figure 1:**
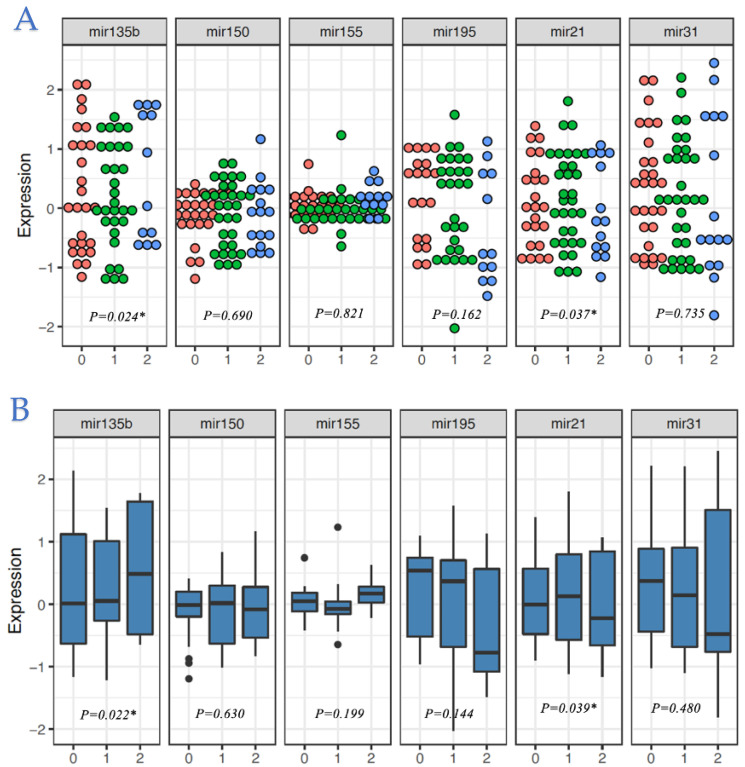
Dot plots and box plots displaying distribution of (**A**) median and (**B**) mean expression levels of the 6 target miRNAs within each nodal stage. * Denotes statistical significance at level *p* < 0.050.

The mean and median expression levels of six target miRNAs (miR-21, miR-31, miR-135b, miR-150, miR-155, and miR-195) and significance tests of differences in distributions for subpopulations, by histopathological tumour stage, nodal stage, EMVI, LVI, location, differentiation, and histology are provided in Table 3. Significant differences were observed for nodal stage with miR-135b (*p* = 0.022) and miR-21 (*p* = 0.039) (Figure 1), with increased expression levels of miR-135b correlated with increased degree of nodal burden and decreased expression levels of miR-21 correlated with increased degree of nodal burden (Figure 1). MiRNA expression profiles and their associations with other clinicopathological characteristics are outlined in the Supplementary Figures S1–S5.

### 3.3. MiRNA as Biomarkers of Colorectal Cancer Recurrence

In differentiating between groups of patients by disease recurrence, miR-135b and miR-195 displayed significant differences in distributions (*p* = 0.023 and *p* = 0.006, respectively) (Table 3). Using univariable Cox regression analysis, reduced expression of miR-135b (β-coefficient −1.126, hazard ratio 0.324, standard error (SE) 0.4698, *p* = 0.017) and increased expression of miR-195 (β-coefficient 1.442, hazard ratio 4.229, SE 0.446, *p* = 0.001) were significant in predicting time to disease recurrence. Using multivariable analysis, miR-195 (β-coefficient 3.187, hazard ratio 24.210, SE 1.419, *p* = 0.025) independently predicted time to CRC recurrence (Table 4). 

ROC analysis was then performed to outline the predictive value of miR-135b and miR-195 in identifying patients likely to suffer disease recurrence following primary colorectal cancer diagnosis. The highest AUC generated from the ROC curve analysis for miR-195 was 79.1% (95% CI: 68.4–89.8%) with a maximum sensitivity of 72% and specificity of 81%, respectively (Supplementary Figure S6A). ROC curve analysis for miR-135b provided a more modest diagnostic test accuracy for predicting recurrence 60.8% (95% CI: 49.1–72.4%) with a maximum sensitivity of 92% and specificity of 34%, respectively (Supplementary Figure S6B). 

**Figure 2:**
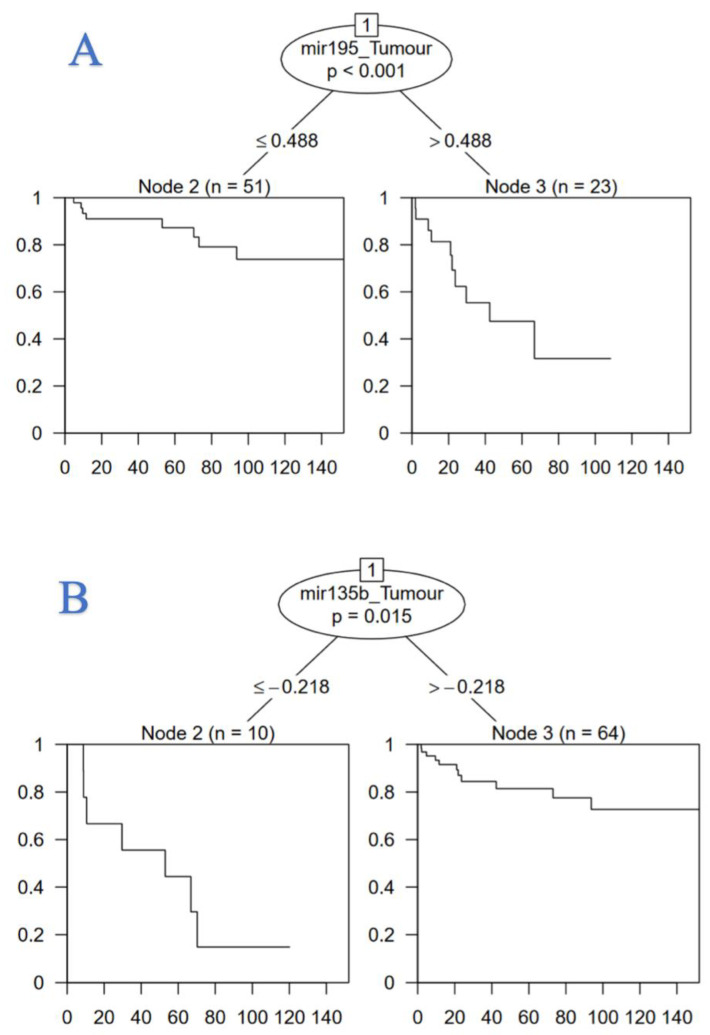
Regression tree illustrating the clinical utility of (**A**) miR-195 and (**B**) miR-135b measurement to predict time to recurrence in analysis of 74 patients treated with curative intent for colorectal carcinoma.

Survival regression classification tree analysis was performed for miR-135b and miR-195. This analysis classified the relevant significant clinical cut-off for miR-195 in modelling time to recurrence in our cohort of 74 patients diagnosed with CRC, where a relative cut-off of ≤0.488 for miR-195 (*p* < 0.001) and a relative cut-off of >−0.218 for miR-135b was associated with improved time to disease recurrence (*p* = 0.015) in CRC patients. (Figure 2). Cox regression and survival regression classification tree analyses with respect to DFS and OS failed to achieve significance and are outlined detail in the Supplementary Figures S7–S8, Table S1–S2. 

## 4. Discussion

The era of precision oncology has facilitated the (de)escalation of cancer therapeutics and surgery where appropriate to ensure treatment planning aligns with maximizing the potential treatment effect to the tumour while minimizing potentially unnecessary toxicities to the patient [42,43]. This encapsulates the ideology of personalised medicine; although, this concept may be criticized for oversimplifying treatment strategies of complex heterogenous diseases such as CRC. The most important finding in this pre-clinical study is the data supporting miR-195 as a predictive biomarker of identifying patients at risk of suffering disease recurrence following curative treatment for CRC. MiR-195 demonstrated a strong diagnostic test accuracy AUC of 79.1% and served as the sole independent predictor of recurrence in our Cox regression and regression tree analyses. This is an important finding, which may be considered unsurprising. MiR-195 is a member of the miR-15/107 family and is renowned as being a stress inducible target, which plays a key oncogenic role in cancer development [21,44]. Moreover, the human miR-195 gene encodes from intron 7 located on chromosome 17p13.1 and on the reverse strand of the mRNA gene AK098506, encoding an unknown hypothetical and functional protein LOC284112 [45,46], which may act as a protein which is key to initiating the cascade responsible for cancer recurrence. Furthermore, this finding correlating miR-195 to disease recurrence becomes clinically relevant when interpreting the results of our regression tree, where relative expression levels of miR-195 greater than or equal to 0.488 indicate an increased recurrence risk at 10-year follow-up. This finding may prove useful to the oncologist in guiding with therapeutic decisions regarding the (de)escalation of treatment in cases of clinical uncertainty and conundrum. Moreover, this is a novel finding, which is uncorroborated by the previous results of a similar analysis. Yang et al., reported reduced miR-195 expression predicted patients likely to succumb to early CRC recurrence (*p* = 0.040) [47]. These results fall short of refuting our findings, as their ROC analysis illustrates only moderate prognostic accuracy of miR-195 in deciphering patients likely to suffer early recurrence in their study (Yang-AUC 61.5% vs. Davey-AUC 79.1%). Of note, other reports cast uncertainty over the biomolecular and cellular role of miR-195 within the setting of CRC, with data supporting miR-195’s role in the modulation of tumour proliferation, facilitating epithelial-to-mesenchymal transition, and, similar to our analysis, propagating metastatic dissemination [44,48,49,50]. 

In this analysis, miR-21 and miR-135b expression levels correlated with the degree of disease burden in the locoregional lymph nodes at resection. These are very interesting findings. The capacity of miR-21 to serve reliably as an oncogenic miRNA has been well described in the oncological literature, with miR-21 being associated with oncogenesis in several malignancies including breast, oesophageal, colorectal, and non-small cell lung carcinoma [51,52,53,54]. These oncogenic properties translate directly to poorer oncological and survival outcomes. In a meta-analysis of 1654 cancer patients, increased miR-21 expression correlated to worse OS in circulation (hazard ratio (HR) 2.37), which was increased exponentially when quantified in gastrointestinal cancer patients (HR 5.77) [55]. In their analysis of 105 pair-matched CRC tumour and ‘normal’ epithelium tissue specimens, Wu et al., previously correlated increased miR-21 expression with advanced CRC staging, lymph node metastases, local invasion, and increased serum carcinoembryonic antigen (CEA) levels through inhibition of the PTEN tumour suppressor gene [56]. The correlation between miR-21 expression and increased nodal status has been reported in human epidermal growth factor receptor-2 (HER2) positive breast cancers, gastric carcinoma, and CRC [57,58,59]. These findings are somewhat unsurprising. In the human genome, coding for miR-21 is located on chromosome 17q23.2. Amplification of the WIP1 gene at 17q23 occurs in 11–18% of cancers [60,61], a large proportion of which tend to be clinically aggressive cancers and harbour HER2 positivity [62,63,64]. Thus, the correlation between increased miR-21 expression and aggressive histopathological features such as increased disease burden in the regional lymph nodes is intelligible. Of note, HER2 fluorescence in situ hybridization assessment was not performed on any of the 74 included CRC patients in this study, although there is data indicating that between 2.5–26.7% of CRC harbour HER2 positivity [65,66]. Huang et al., previously illustrated that miR-21 is upregulated via the MAPK signalling pathway through HER2 signalling [67], which perhaps is unsurprising when considering the proximity of the miR-21 gene, the WIP1 gene, and the HER2 amplicon at 17q23. Nevertheless, we must reiterate that this analysis adds to the evidence illustrating the relationship with miR-21 expression levels and nodal stage in malignancy [57,58,59].

As observed with miR-21 expression, increased miR-135b expression correlated with nodal status in the current study. MiR-135b is encoded in the LEMD1 gene located at 1q32.1 and is known to be aberrantly expressed in CRC progression [68,69]. Liu et al., hypothesize that increased miR-135b expression promotes tumour progression through targeting the transforming growth factor beta receptor 2 (TGFBR2) [70], which is known to play a key role in cellular processes, including cell cycle regulation, apoptosis, and immune modulation [71]. In previous translational research studies, miR-135b has been amplified as an oncogenic miRNA in the setting of colorectal cancer [72], with several studies demonstrating the ability of miR-135b to differentiate tumour from both adenoma and ‘normal’ epithelial tissues [73,74]. Interestingly, Wu et al., illustrated the regulatory role of miR-135b on metastases suppressor-1 expression profiles in their analysis of 113 samples [75]. In their study, increased miR-135b expression promoted locoregional and distant disease metastases, supporting the data correlating miR-135b expression with nodal stage as well as the preliminary data associating the biomarker with recurrence risk in our univariable analysis. Nagel et al., previously outlined the tumour suppressor role of miR-135b in regulating Adenomatous Polyposis Coli Gene expression levels [69], while Sarver et al., illustrate the impact of miR-135b concentrations in modulating mismatch repair status in advanced disease [74]. When translating the correlation of miR-135b with nodal staging to clinical practice, this remains an interesting and important surgical finding. In the AJCC 8th edition for CRC tumour, nodes, metastases (TNM) staging system, nodal status is staged in stepwise classification as N0, N1a, N1b, N2a, and N2b based on the number of positive dissected lymph nodes (0, 1, 2–3, 4–6, more than 7) due to their predictive power in anticipating outcome in CRC [28,76]. Lymph node involvement in CRC now forms the cornerstone of staging locally advanced CRC tumours as AJCC staging focuses on the presence/absence of invasive cancer in locoregional lymph nodes to delineate stage II and stage III disease, with those with even one node involved automatically being classified as stage IIIa or above at histopathological evaluation [28]. Real-world data support this modification to AJCC CRC staging. In their meta-analysis of 33,984, Bockelman et al., reported the obvious survival advantage for 15,559 patients with locally advanced stage II CRC compared with their 18,425 counterparts with stage III disease (5-year DFS in receipt of adjuvant chemotherapy: 81.4% vs. 49.0%, 5-year DFS without adjuvant chemotherapy: 79.3% vs. 63.6%) [11]. Thus, it is imperative that investigations informing nodal status remains at the epicentre of translational research efforts to inform prognoses and personalise therapeutic decision making to complement the patient and their disease profile. 

While this analysis provides novel insights into the clinical role of three previously described miRNA, we must acknowledge the limitations of the current biomarker discovery study. While the detection of miR-195 is promising in predicting disease recurrence, its relevance is somewhat limited by just ‘acceptable’ diagnostic test accuracy in a non-cross validated model [77]. Our attempts to strengthen the accuracy of this analysis through the inclusion miR-135b was unsuccessful due to miR-135b’s modest diagnostic test accuracy results in predicting recurrence. Perhaps, this clinical utility of miR-195 may be strengthened through its inclusion in a multi-miRNA expression assay with other more sensitive targets. Additionally, this study fails to consider the amplification and validation of miRNA assays in patient circulation, which may be perceived as a clinically important next step before considering its use as a biomarker for detecting recurrence. Furthermore, this study was conducted in a single translational research centre, where recruited patients represent a culturally unique Irish population, implying there is a possibility of limited genetic diversity and relative homogeneity of patients. Moreover, this analysis includes 22 patients treated with curative intent for invasive rectal carcinoma, yet only 12 patients received neoadjuvant therapies. Since recruiting the earlier patients to this study, the paradigm has evolved significantly to recognise the inherent value of the multimodal use of combined chemoradiotherapy in the neoadjuvant setting as the ‘gold standard’ for rectal carcinoma [78]. Therefore, this study may be accused of being culpable of pooling all 74 patients under the umbrella term ‘CRC’ and failing to highlight the different molecular subtypes, therapeutic strategies, and surgical approaches applied to these independent subgroups in this cohort. In spite of these limitations, the current analysis provides novel clinically relevant molecular biomarkers capable of substratifying CRC patients into those at an increased risk of disease recurrence, adding to the modern convention to personalise our managerial approach for prospective CRC patients.

## 5. Conclusions

In conclusion, this study comprehensively illustrates the prognostic value of miR-195 in predicting recurrence in a cohort of 74 patients being treated curatively for CRC. Furthermore, this analysis highlights the clinically relevant cut-offs that predict poorer outcomes for those with increased miR-195 expression. Additionally, the results of this study support measurement of miR-21 and miR-135b as useful biomarkers to predict the degree nodal burden at the time of resection. Future studies may validate these novel findings to facilitate the personalisation of therapeutic strategies for patients being treated for CRC.

## Figures and Tables

**Table 1:** Patient demographics, clinicopathological, and survival data for the 74 patients with colorectal cancer included in this study.

Clinicopathological Parameter	Patients with Colorectal Cancer (N = 74)
**Mean age** (±SD, range)	67.8 years (±12.5, 38–90 years)
**Gender** Male Female	51 (68.9%) 23 (31.1%)
**Tumour Location** Colon Rectum	52 (70.3%) 22 (29.7%)
**Presentation** Emergency Elective	12 (16.2%) 62 (83.8%)
**Histological subtype** Adenocarcinoma Mucinous Other/Missing	53 (68.0%) 3 (3.8%) 22 (28.2)
**Tumour Stage** T1 T2 T3 T4 TX	2 (2.7%) 5 (6.8%) 25 (33.8%) 18 (24.3%) 24 (32.4%)
**Nodal Stage** N0 N1 N2 NX	15 (20.3%) 20 (27.0%) 10 (13.5%) 29 (39.2%)
**Chemotherapy** Received NAC Received AC Did not receive/Unknown	12 (16.2%) 40 (54.1%) 22 (29.7%)
**Recurrence** Recurrence No Recurrence	18 (24.3%) 56 (75.7%)
**Recurrence Location** Liver Lung Adrenal Liver and Lung Adrenal and Liver Mediastinum Pelvis None	7 (9.0%) 3 (3.9%) 2 (2.6%) 1 (1.3%) 1 (1.3%) 1 (1.3%) 3 (3.9%) 56 (75.7%)
**Survival** Alive RIP	43 (58.1%) 31 (41.9%)
**5-Year DFS**	51/74 (68.9%)
**5-Year OS**	53/74 (71.6%)

SD—standard deviation, NAC—neoadjuvant chemotherapy, AC—adjuvant chemotherapy, RIP—rest in peace, DFS—disease-free survival, OS—overall survival.

**Table 2:** Brief descriptions of the relevance of the 6 target miRNA and 2 endogenous controls in the setting of colorectal and other carcinoma.

Target	MiRNA Function	Expression Levels	CT Difference	Efficiencies
**miR-21**	Well-described oncogenic miRNA in several malignancies [21]	Increased	12.19	97%
**miR-31**	Previously reported oncogenic miRNA in CRC [33]	Increased	14.42	101%
**miR-135b**	Modulatory role in malignancy and CRC [34]	Decreased	14.13	99%
**miR-150**	Associated with disease progression and metastases in CRC [35]	Increased	10.88	106%
**miR-155**	Correlated to CRC development, invasion, and metastasis [36]	Increased	13.83	108%
**miR-195**	Known oncogenic biomarker in malignancy [21]	Increased	11.88	93%
**miR-16**	Endogenous control [37]	Stable	0.00	-
**miR-345**	Endogenous control [37]	Stable	0.00	-

CT—cycle threshold, CRC—colorectal cancer.

**Table 3:** Correlation of miRNA expression profiled with clinicopathological, recurrence, and survival data.

Parameter	Comparing Means					
	miR-21	miR-31	miR-135b	miR-150	miR-155	miR-195
**Tumour Stage** ▯	0.399	0.561	0.509	0.079	0.008 *	0.308
**Nodal Stage** ▯	0.037 *	0.735	0.024 *	0.690	0.821	0.162
**EMVI** ▯	0.957	0.349	0.860	0.456	0.507	0.029 *
**LVI** ▯	0.899	0.249	0.341	0.982	1.000	0.748
**Colon vs. Rectal** ▯	0.850	0.035 *	0.284	0.052	0.272	0.327
**Differentiation** ▯	0.887	0.275	0.307	0.051	0.179	0.532
**Histology** ▯	0.224	0.083	0.629	0.045 *	0.048 *	0.118
**Recurrence** ▯	0.857	0.550	0.013 *	0.538	0.602	0.001 *
**Mortality** ▯	0.431	0.800	0.980	0.955	0.979	0.609
**Parameter**	**Comparing Medians**					
	**miR-21**	**miR-31**	**miR-135b**	**miR-150**	**miR-155**	**miR-195**
Tumour Stage †	0.365	0.652	0.628	0.103	0.139	0.226
Nodal Stage †	0.039 *	0.480	0.022 *	0.630	0.199	0.144
EMVI †	0.889	0.711	0.889	0.667	0.500	0.095
LVI †	0.141	0.221	0.781	0.891	0.233	0.256
Colon vs. Rectal †	0.614	0.068	0.393	0.024 *	0.757	0.199
Differentiation †	0.889	0.581	0.222	0.183	0.222	0.889
Histology †	0.424	0.078	0.547	0.053	0.051	0.100
Recurrence †	0.354	0.677	0.023 *	0.370	0.818	0.006 *
Mortality †	0.386	0.930	0.831	0.889	0.791	0.934

▯ Denotes Independent Student’s T-test. † Denotes Kruskal—Wallis test. * Denotes statistical significance at level *p* < 0.050.

**Table 4:** Binary logistic and Cox regression analyses to determine predictors in modelling time to recurrence.

Binary Outcome-Recurrence					Cox Regression-Recurrence			
Parameter	β-Coefficient (SE)	*p*-Value	β-Coefficient (SE)	*p*-Value	*HR (95% CIs)*	*p*-Value	*HR (95% CIs)*	*p*-Value
	Univariable		Multivariable		Univariable		Multivariable	
**miR-21**	0.282 (0.579)	0.626	0.372 (1.138)	0.744	1.303 (0.430–4.130)	0.626	1.450 (0.160–13.490)	0.744
**miR-31**	0.403 (0.413)	0.330	−0.125 (0.770)	0.873	1.500 (0.670–3.360)	0.330	0.880 (0.200–3.990)	0.871
**miR-135b**	−1.126 (0.467)	0.017 *	−0.515 (0.808)	0.524	0.320 (0.130–0.810)	0.017 *	0.600 (0.120–2.910)	0.524
**miR-150**	1.156 (0.630)	0.067	1.023 (1.040)	0.325	3.180 (0.920–0.91)	0.067	2.780 (0.360–21.340)	0.325
**miR-155**	−0.016 (0.702)	0.982	3.175 (2.139)	0.138	0.980 (0.250–3.900)	0.982	23.910 (0.360–21.340)	0.138
**miR-195**	1.442 (0.446)	0.001 *	3.187 (1.419)	0.025 *	4.230 (1.77–10.13)	0.001 *	24.210 (1.500–390.780)	0.025 *

SE—standard error, HR—hazard ratio, CI—confidence interval. * Denotes statistical significance.

## Data Availability

Data can be obtained upon reasonable request from the corresponding author.

## Supplementary Materials

The following supporting information can be downloaded at: https://www.mdpi.com/article/10.3390/cancers14092109/s1, Figure S1. Dotplots and Boxplots displaying distribution of the (A) median and (B) mean expres-sion levels of the 6 target miRNAs by tumour stage. Figure S2. Dotplots and Boxplots displaying distribution of the (A) median and (B) mean expres-sion levels of the 6 target miRNAs by site of primary cancer. Figure S3. Dotplots and Boxplots displaying distribution of the (A) median and (B) mean expres-sion levels of the 6 target miRNAs by the presence of extramural vascular invasion. Figure S4. Dotplots and Boxplots displaying distribution of the (A) median and (B) mean expres-sion levels of the 6 target miRNAs by the degree of differentiation. Figure S5. Dotplots and Boxplots displaying distribution of the (A) median and (B) mean expres-sion levels of the 6 target miRNAs by histological subtype. Figure S6. Receiver operating characteristic curve using logistic regression analysis to illustrate the accuracy of miR-195 (A) and miR-135b (B) in predicting recurrence in colorectal cancer by logistic regression. Figure S7. Regression descriptive classification tree illustrating the clinical utility of miR-21 meas-urement to differentiate time to overall survival mortality in an analysis of 58 patients treated with curative intent for colorectal carcinoma. Figure S8. Regression descriptive classification tree illustrating the clinical utility of miR-21, miR-31 and miR-135b measurement in differentiating disease free survival time in an analysis of 68 patients treated with curative intent for colorectal carcinoma. Table S1. Binary logistic and Cox-regression analyses to determine predictors in modelling overall survival time. Table S2. Binary logistic and Cox-regression analyses to determine predictors in modelling disease-free survival.
