# Supplementary material for: MicroRNA Expression Profiling Predicts Nodal Status and Disease Recurrence in Patients Treated with Curative Intent for Colorectal Cancer
Source: Cancers (Basel). 2022 Apr 23;14(9):2109. doi: 10.3390/cancers14092109 (PMC9106021; doi:10.3390/cancers14092109)
Supplement: Supplementary file 1 [file cancers-14-02109-s001.zip › cancers-1665466-supplementary.pdf]

Supplementary Material

MicroRNA Expression Profiling Predicts Nodal Status and Disease Recurrence in Patients Treated with Curative Intent for Colorectal Cancer

Matthew G. Davey, Gerard Feeney, Heidi Annuk, Maxwell Paganga, Emma Holian, Aoife J. Lowery, Michael J. Kerin, Nicola Miller

[1] Department of Surgery, Lambe Institute for Translational Research, National University of Ireland, Galway, Galway, Republic of Ireland.

[2] Department of Statistics and Mathematics, National University of Ireland, Galway, Galway, Republic of Ireland.

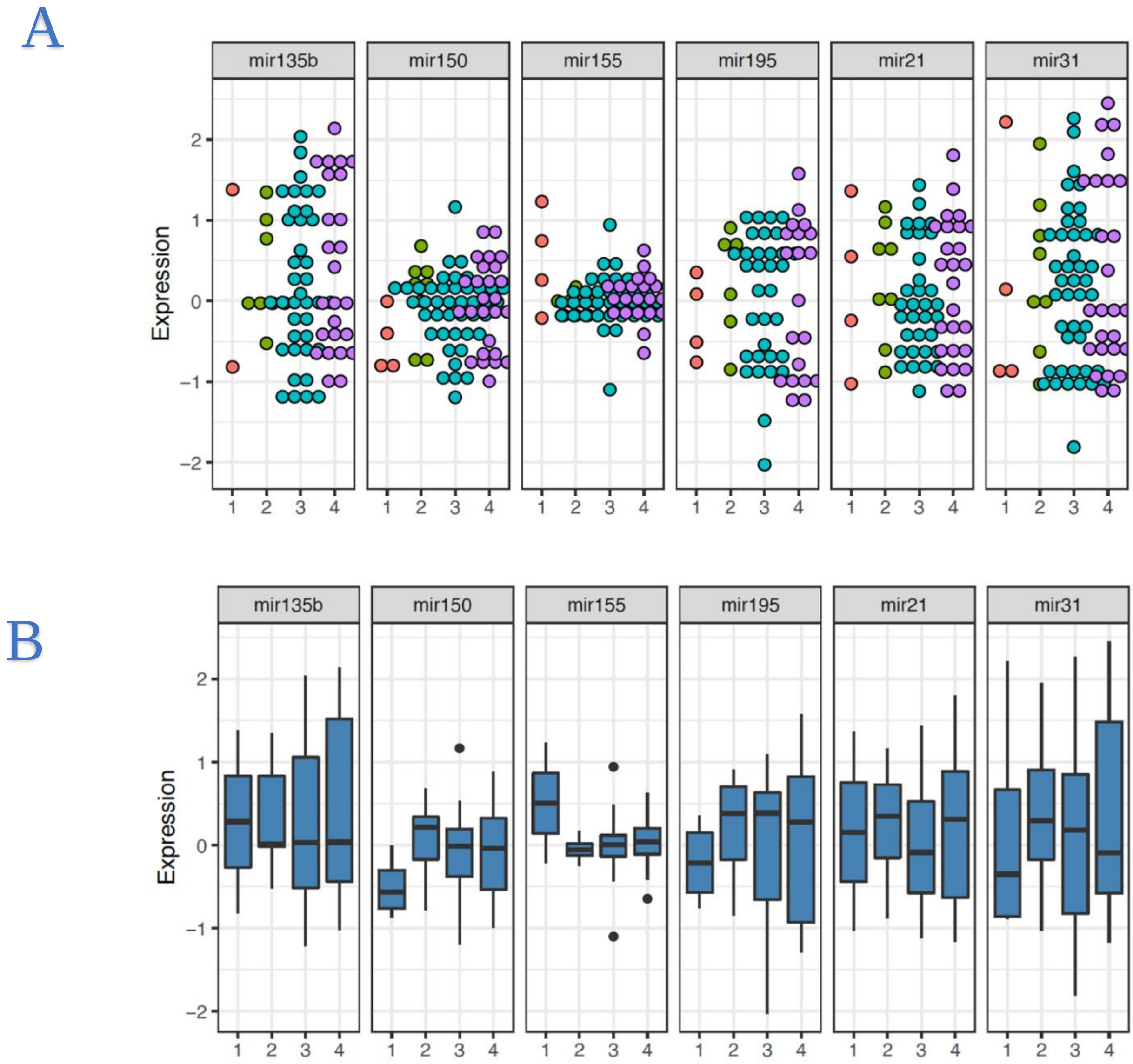

Figure S1. Dotplots and Boxplots displaying distribution of the (**A**) median and (**B**) mean expression levels of the 6 target miRNAs by tumour stage.

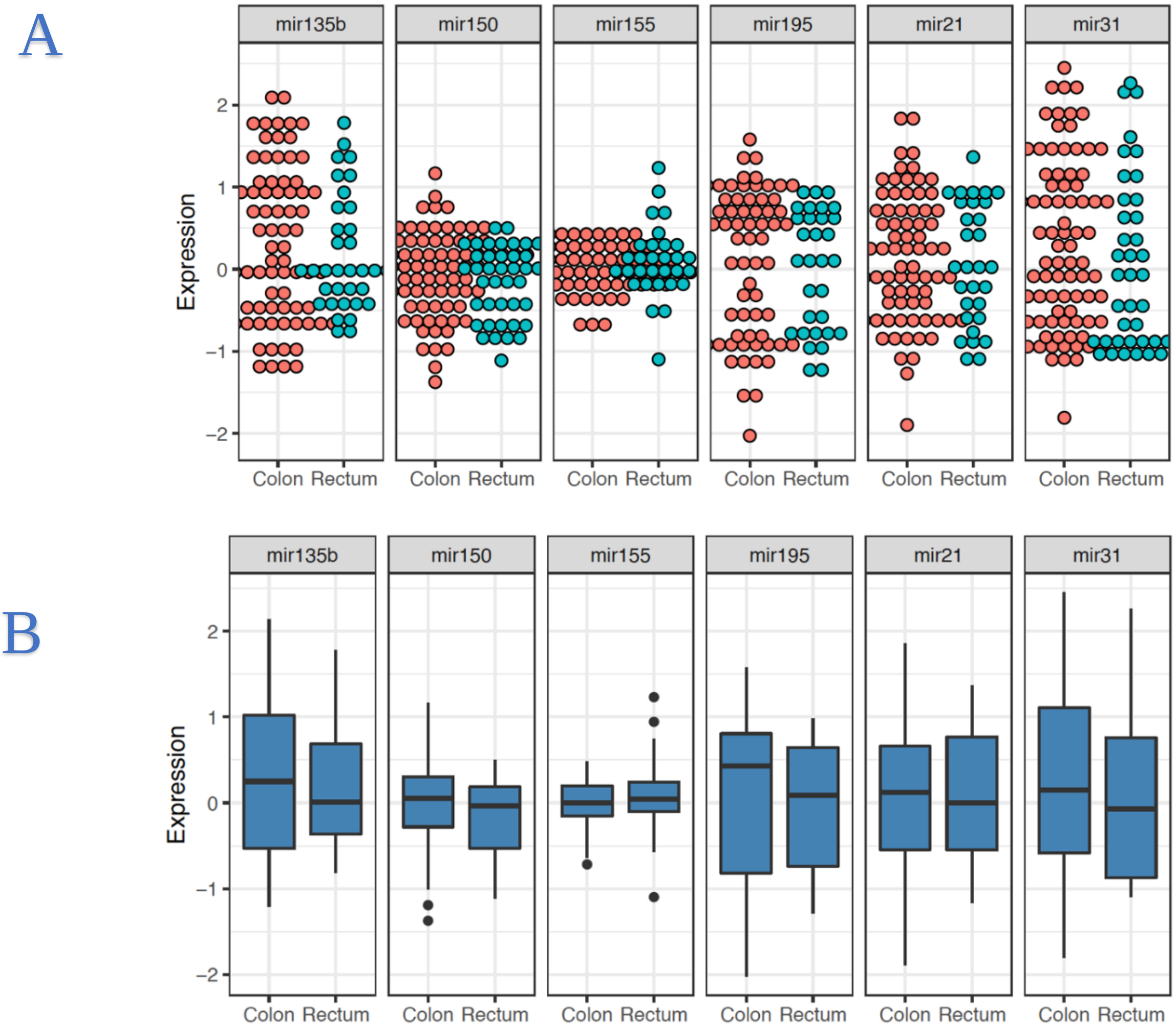

Figure S2. Dotplots and Boxplots displaying distribution of the (**A**) median and (**B**) mean expression levels of the 6 target miRNAs by site of primary cancer.

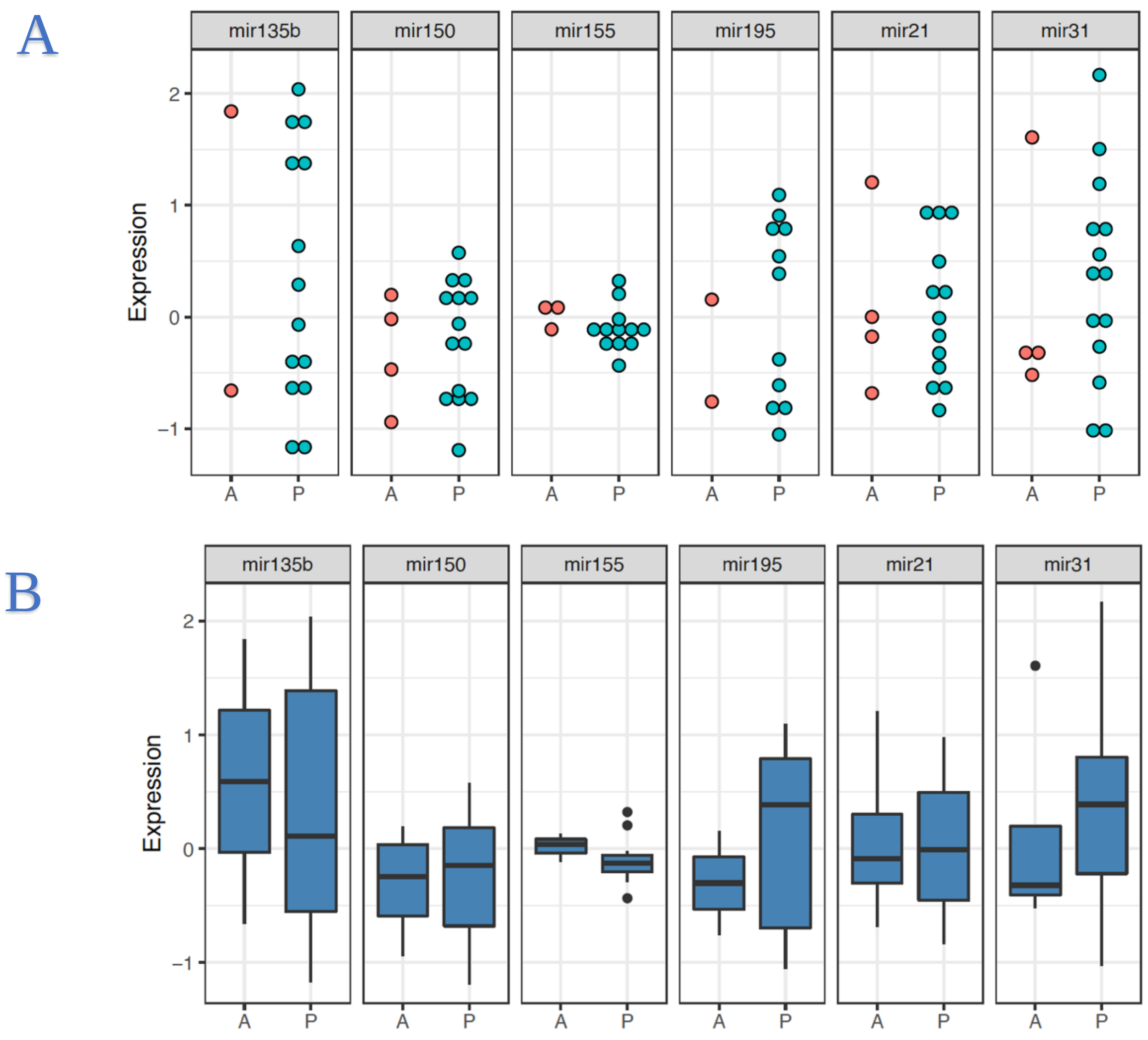

Figure S3. Dotplots and Boxplots displaying distribution of the (**A**) median and (**B**) mean expression levels of the 6 target miRNAs by the presence of extramural vascular invasion.

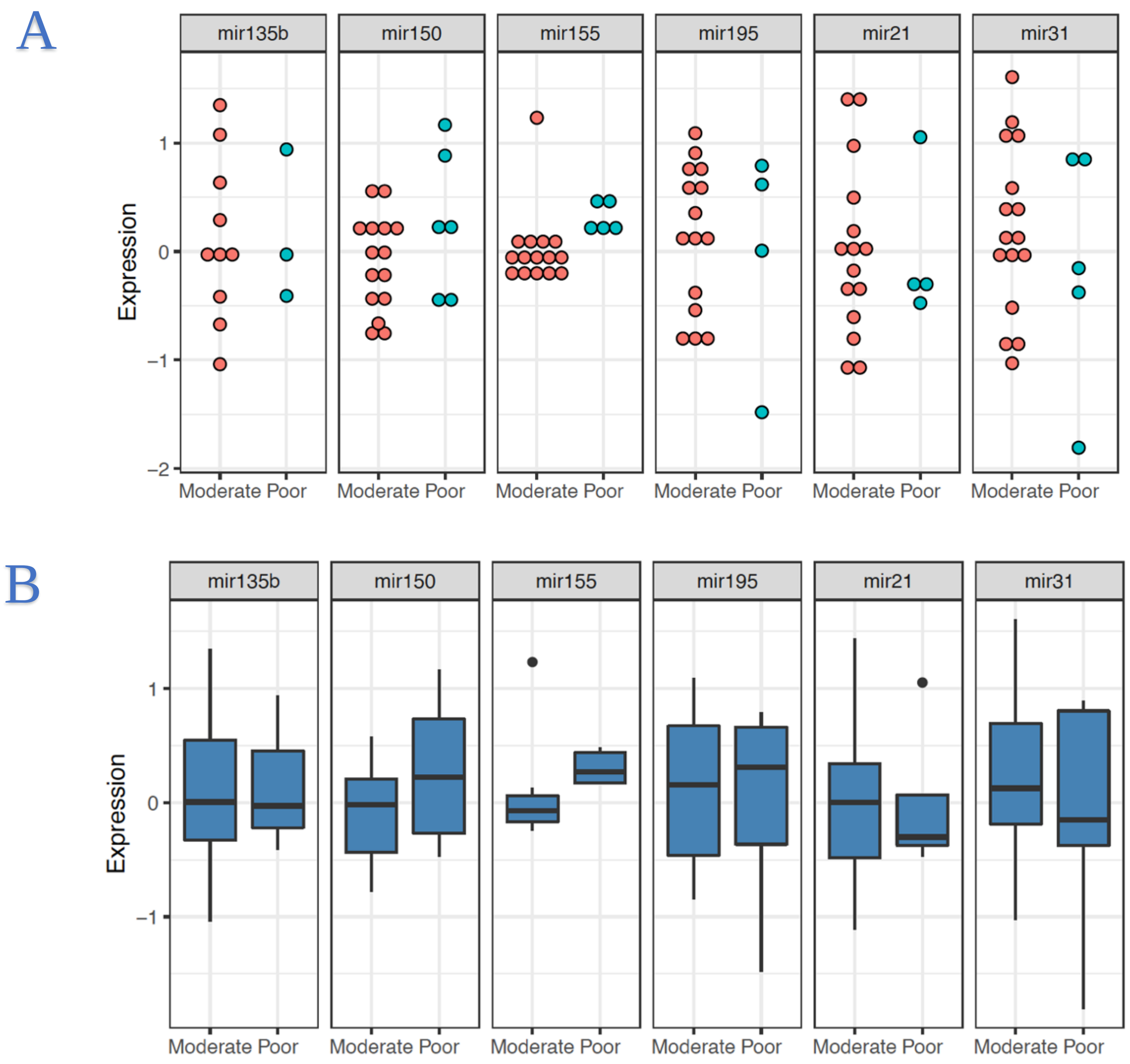

Figure S4. Dotplots and Boxplots displaying distribution of the (**A**) median and (**B**) mean expression levels of the 6 target miRNAs by the degree of differentiation.

.

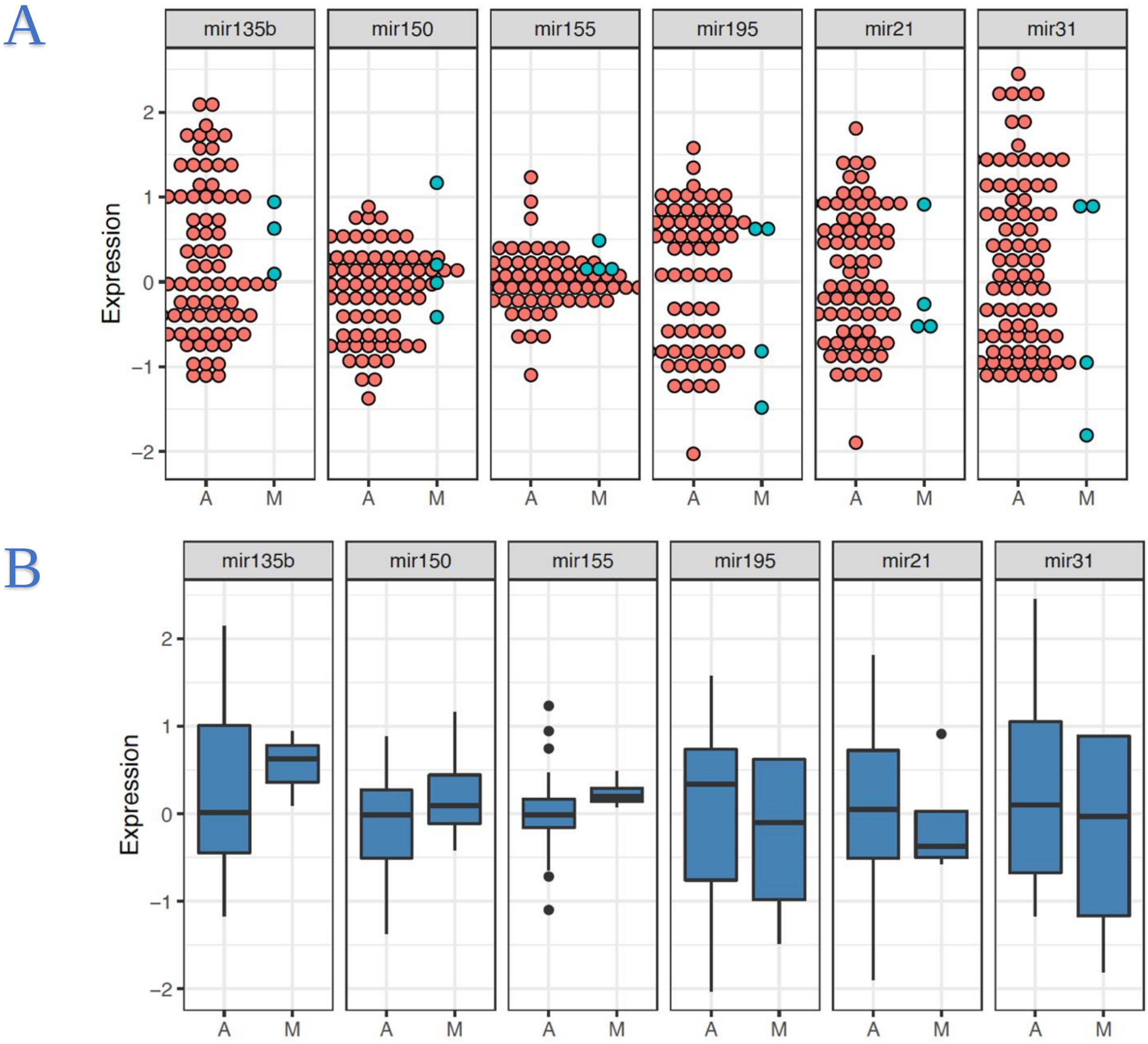

Figure S5. Dotplots and Boxplots displaying distribution of the (**A**) median and (**B**) mean expression levels of the 6 target miRNAs by histological subtype.

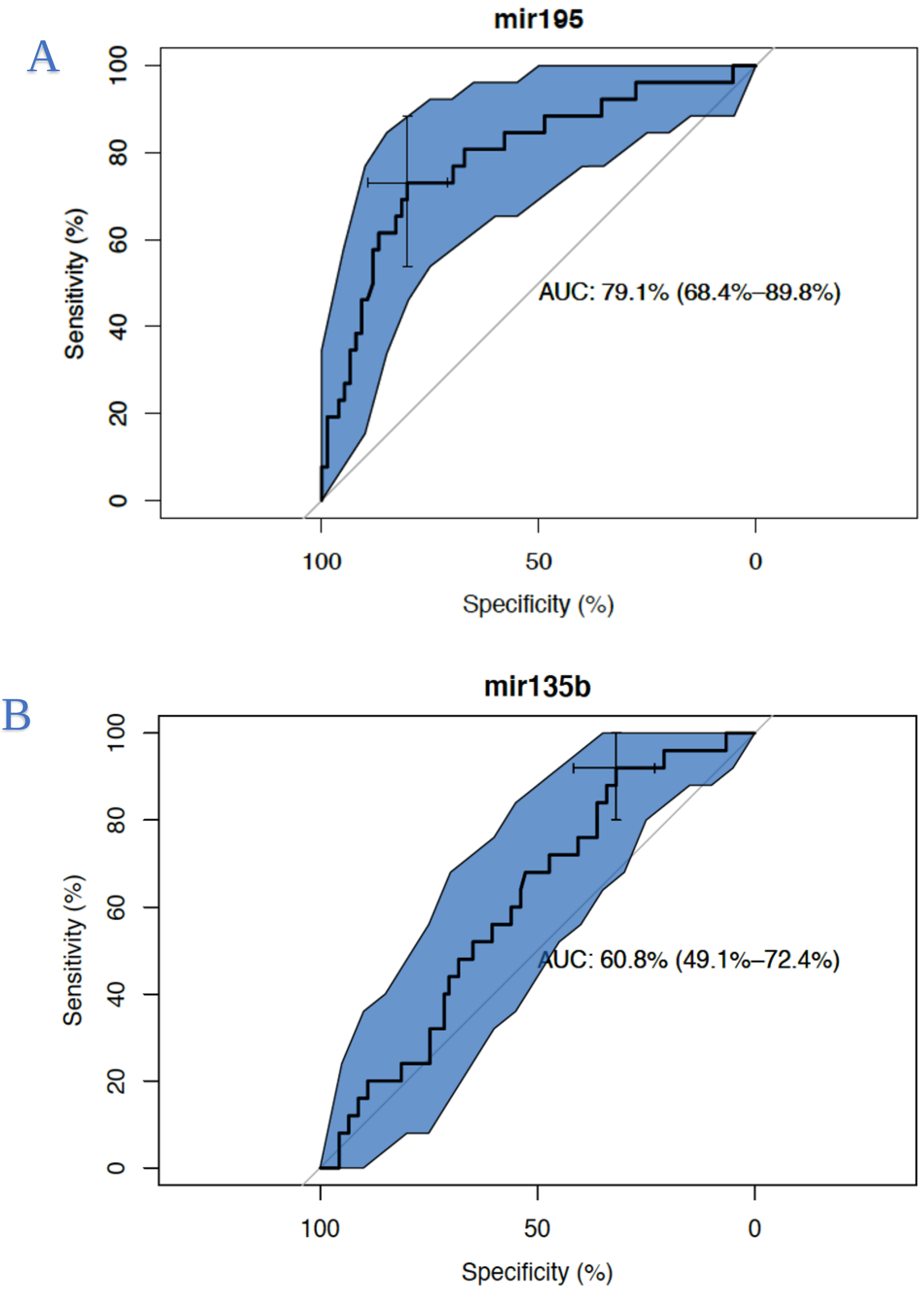

Figure S6. Receiver operating characteristic curve using logistic regression analysis to illustrate the accuracy of miR-195 (**A**) and miR-135b (**B**) in predicting recurrence in colorectal cancer by logistic regression.

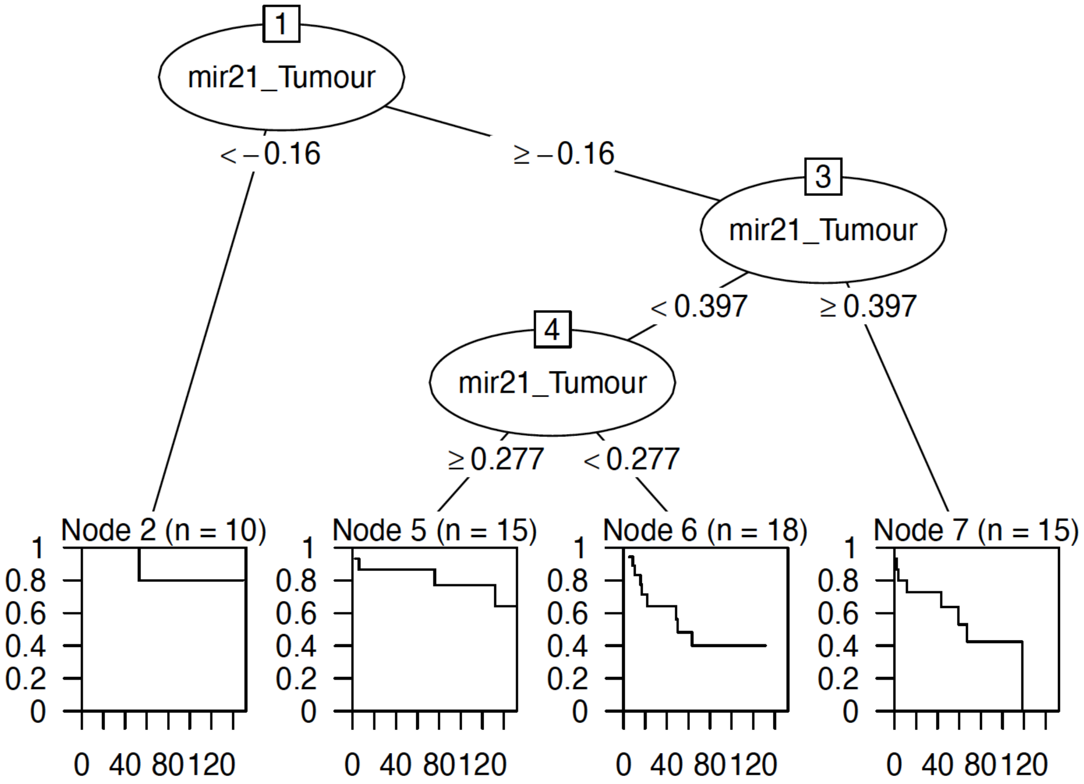

Figure S7. Regression descriptive classification tree illustrating the clinical utility of miR-21 measurement to differentiate time to overall survival mortality in an analysis of 58 patients treated with curative intent for colorectal carcinoma.

Table S1. Binary logistic and Cox-regression analyses to determine predictors in modelling overall survival time.

	Binary Outcome Overall Survival.				Cox Regression—Overall Survival			
**Parameter**	**Coefficient (SE) Univariable**	***P*-value**	**Coefficient (SE) Multivariable**	***P*-value**	***HR (95% CIs)* Univariable**	***P*-value**	***HR (95% CIs)* Multivariable**	***P*-value**
**miR-21**	0.477 (0.492)	0.333	–0.221 (1.007)	0.827	1.610 (0.610–4.230)	0.333	0.802 (0.111–5.772)	0.827
**miR-31**	0.113 (0.279)	0.685	–0.700 (0.526)	0.184	1.120 (0.650–1.930)	0.685	1.344 (0.360–5.012)	0.184
**miR-135b**	−0.082 (0.354)	0.816	–0.422 (0.596)	0.478	0.920 (0.460–1.840)	0.816	0.497 (0.177–1.393)	0.478
**miR-150**	0.390 (0.442)	0.375	0.747 (0.777)	0.337	1.480 (0.620–3.520)	0.375	0.655 (0.204–2.107)	0.337
**miR-155**	–0.241 (0.546)	0.660	–0.625 (0.965)	0.517	0.790 (0.270–2.290)	0.660	2.110 (0.460–9.672)	0.517
**miR-195**	0.479 (0.389)	0.219	0.295 (0.672)	0.660	1.610 (0.750–3.460)	0.219	0.535 (0.080–3.545)	0.660

SE; standard error, HR; hazard ratio, CI; confidence interval. *Denotes statistical significance.

Table S2. Binary logistic and Cox-regression analyses to determine predictors in modelling disease-free survival.

	Binary Outcome Disease-Free Survival				Cox Regression - Disease-Free Survival			
**Parameter**	**Coefficient (SE) Univariable**	***P*-value**	**Coefficient (SE) Multivariable**	***P*-value**	***HR (95% CIs)* Univariable**	***P*-value**	***HR (95% CIs)* Multivariable**	***P*-value**
**miR-21**	*0.445 (0.440)*	*0.312*	–0.318 (0.900)	0.724	1.560 (0.660–3.700)	*0.312*	*0.728 (0.125–4.247)*	0.724
**miR-31**	*0.143 (0.265)*	*0.589*	–0.485 (0.453)	0.285	1.150 (0.690–1.940)	*0.589*	*2.301 (0.583–9.091)*	0.285
**miR-135b**	*–0.240 (0.337)*	*0.477*	–0.258 (0.571)	0.651	0.790 (0.410–1.520)	*0.477*	*0.616 (0.253–1.497)*	0.651
**miR-150**	*0.287 (0.453)*	*0.526*	0.182 (0.718)	0.801	1.330 (0.550–3.240)	*0.526*	*0.772 (0.252–2.367)*	0.801
**miR-155**	*0.106 (0.522)*	*0.839*	0.591 (0.830)	0.478	1.111 (0.400–3.090)	*0.839*	*0.834 (0.293–4.901)*	0.478
**miR-195**	*0.557 (0.357)*	*0.119*	0.834 (0.701)	0.234	1.750 (0.870–3.510)	*0.357*	*0.553 (0.353–9.239)*	0.234

SE; standard error, HR; hazard ratio, CI; confidence interval. *Denotes statistical significance.

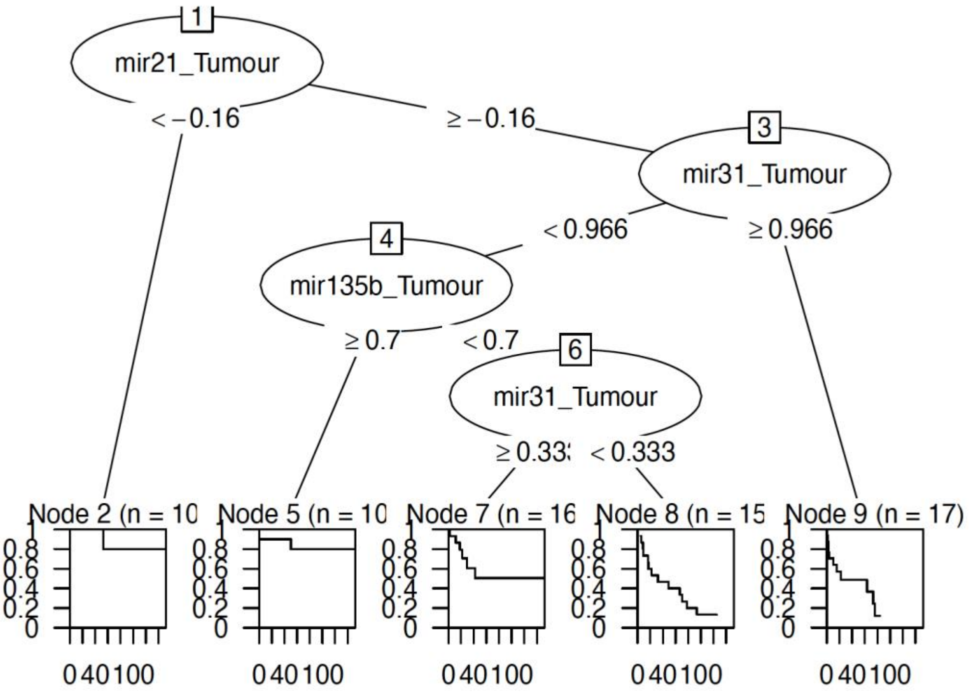

Figure S8. Regression descriptive classification tree illustrating the clinical utility of miR-21, miR-31 and miR-135b measurement in differentiating disease free survival time in an analysis of 68 patients treated with curative intent for colorectal carcnoma.
